# *GhA01EP1* of Upland Cotton Stimulates Precocity, Improved Water Deficit Tolerance, and High Seed Yield in Transgenic *Arabidopsis*

**DOI:** 10.3390/genes16060669

**Published:** 2025-05-30

**Authors:** Dan Li, Cunpeng Zhao, Xiaohui Zhang, Haina Zhang, Chen Yuan, Kaihui Wang, Suen Liu, Junyi Geng, Baosheng Guo

**Affiliations:** 1 Key Laboratory of Cotton Biology and Genetic Breeding in Huanghuaihai Semiarid Area, Ministry of Agriculture and Rural Affairs, Institute of Cotton, Hebei Academy of Agriculture and Forestry Sciences, Shijiazhuang 050051, China; lidan@haafs.org (D.L.); zhaocunpeng@haafs.org (C.Z.); zhanghaina@haafs.org (H.Z.); 18222060565@163.com (C.Y.); wangkaihui@haafs.org (K.W.); liusuen@haafs.org (S.L.); gengjunyi@haafs.org (J.G.); 2 College of Food Science and Biology, Hebei University of Science and Technology, Shijiazhuang 050018, China; 3 School Landscape and Ecological Engineering, Hebei University of Engineering, Handan 056038, China

**Keywords:** glycoprotein, precocity, water stress, high seed yield, functional analysis

## Abstract

Background: The *GhA01EP1* gene in upland cotton encodes an epidermal-specific secreted glycoprotein, whose functional characterization remains unexplored beyond our initial discovery of its water deficit resistance association. Therefore, we further designed experiments to investigate the functional role of *GhA01EP1*. Methods: We sequenced and analyzed the transcriptomes of wild-type (Col-0) and *GhA01EP1*-transgenic *Arabidopsis thaliana*. The differences in morphological and biochemical indicators were examined. In addition, the proteins interacting with *GhA01EP1* in *Arabidopsis* were screened using a glutathione-S-transferase pull-down assay. Results: The *GhA01EP1*-transgenic *Arabidopsis* plants flowered earlier, produced more branches, and had a higher seed yield than Col-0. Transcriptome analysis revealed that differentially expressed genes detected in the comparison of *GhA01EP1*-transgenic and Col-0 *Arabidopsis* under the water treatment (the control) were associated especially with circadian rhythm regulation, photoperiodic flowering reaction, hormone metabolism, glyoxalase I synthesis, antioxidant pathway, branching development, and carbon-nitrogen allocation. Under water-sufficient or water-deficient treatments, the glyoxalase I activity and lignin content of *GhA01EP1*-transgenic *Arabidopsis* were significantly higher. Under water deficit stress, the malondialdehyde and starch contents were significantly lower, while peroxidase activity and protein content were significantly higher than those of Col-0. Conclusions: *GhA01EP1* synergistically improved the precocity, water deficit tolerance, and seed yield of *GhA01EP1*-transgenic *Arabidopsis*. Analysis of *GhA01EP1* function provides a molecular basis for breeding improved cotton varieties.

## 1. Introduction

Secretory proteins play an important role in the regulation of physiological activities as carriers of information, material, and energy exchange between plants and the external environment. Studies have shown that secretory proteins perform crucial biological functions in extracellular/intracellular signal transduction, cell response to environmental stimuli, cell wall structure formation, pathogen defense, and cell–cell interaction [1]. More specifically, the plant secretome provides multipronged protection through the perception of cell wall degradation products [2].

The functions of secretory proteins have been widely investigated. For example, hydroxyproline-rich glycoproteins (HRGP), among the most abundant structural proteins in plant cell walls, play critical roles in cell wall-mediated signaling cascades, stress tolerance, and cell differentiation. Arabinogalactan proteins (AGPs), as highly glycosylated members of the HRGP superfamily, are biosynthesized in diverse plant species [3,4]. Functionally, AGPs exhibit pleiotropic regulatory roles, including seedling growth [5], cell division [6], mechanical injury [7], programmed cell death [8], and abiotic stress response [9]. In addition to HRGPs, the functions of other secretory proteins have been analyzed. For instance, members of the pathogenesis-related protein 1 (PR1) family [10] and aspartic proteases [11] are functionally associated with pathogen resistance mechanisms.

The *EP1* gene (*epithelial-specific secreted glycoprotein 1*) encodes a glycoprotein specifically secreted by epithelial cells, playing an important role in maintaining epithelial cells’ structure and function. While current literature on *EP1* remains limited, emerging evidence suggests its possible regulatory involvement in plant growth, development processes, and responses to stress [12]. Engelen et al. speculated that *EP1* in carrot may be involved in certain regulatory pathways that ultimately control water flow [13]. Lei et al. reported that *PoEP1* can regulate early flowering in peony [12]. Our previous study showed that the *EP1-like* gene in cotton (*GhA01EP1*) is associated with water deficit tolerance [14]. However, no studies have demonstrated that *GhA01EP1* regulates flowering initiation or other physiological functions. To further investigate its biological roles and preliminarily explore the underlying mechanisms, we designed this experiment.

*GhA01EP1* was previously cloned from Ji 2658 using a homologous cloning method. Quantitative real-time polymerase chain reaction (qRT-PCR) analysis showed that *GhA01EP1* was expressed in cotton roots, stems, and leaves, and was up-regulated under water deficit stress. Subcellular localization analysis indicated that GhA01EP1 is a secreted protein. *GhA01EP1*-transgenic *Arabidopsis* has stronger water deficit tolerance than the wild-type Columbia (Col-0) ecotype [14]. In the study, we sequenced and analyzed the transcriptomes of Col-0 and *GhA01EP1*-transgenic *Arabidopsis* and evaluated the differences in morphological, biochemical indicators. In addition, the proteins interacting with GhA01EP1 in *Arabidopsis* were screened to examine the molecular function of *GhA01EP1*.

## 2. Materials and Methods

### 2.1. Plant Materials and Treatment

The experimental materials used in the study included *Arabidopsis thaliana* ecotype Col-0, *GhA01EP1*-transgenic *Arabidopsis*, and tobacco (*Nicotiana benthamiana*).

### 2.2. Phenotype, Seed Yield, and Protein and Starch Contents Comparison Between Transgenic Lines and Col-0

Col-0 and *GhA01EP1*-transgenic *Arabidopsis* seeds (five T_3_ transgenic lines) were sown in growth medium (vermiculite:nutrient soil = 1:1, *v*/*v*) and then transferred to an incubator for cultivation. The growth environment was maintained at 22 °C with a 16 h/8 h (light/dark) photoperiod. The transgenic lines and Col-0 were grown under the water-sufficient treatment. Throughout the growth process, the development of *Arabidopsis* was monitored at all times, documenting the flowering period of each seedling (when the main stem reaches 0.5 cm in length) and branch number. Following seed maturation, seeds from individual plants were collected and weighed, with fifteen individual plants collected per sample. Seeds from nine randomly selected individual plants per sample were divided into two aliquots for starch and protein content determination. The analysis of protein and starch contents was conducted according to the instructions of the commercial reagent kit (Grace Biotechnology, Suzhou, China). Starch content was determined using the acid hydrolysis method, while protein content was determined using the Bradford Assay.

### 2.3. Root Growth Assay of Transgenic Arabidopsis Under Water Deficit Stress

Col-0 and T_3_ transgenic line (five lines: D1, D2, D4, D7, D8) seeds were surface-sterilized and sown on Murashige and Skoog (MS) medium (22 °C, 16 h light/8 h dark). After full expansion of cotyledons, uniformly grown seedlings were transferred to MS medium containing 0 mmol∙L^−1^, 250 mmol∙L^−1^, or 300 mmol∙L^−1^ D-mannitol, with three biological replicates in total. MS medium plates were placed vertically in growth chambers. Root lengths of Col-0 and transgenic lines were measured after eight days of treatment [14].

### 2.4. Water Deficit Resistance Evaluation of Transgenic Arabidopsis via Drought-Rehydration Method

Col-0 and transgenic *Arabidopsis* seeds were surface-sterilized and germinated on MS medium for one week. Uniformly grown seedlings were then transplanted into soil with 24 plants per line, with three biological replicates in total. After approximately 2–3 weeks of growth, irrigation was withheld to induce water deficit stress. Plant growth status was monitored continuously until severe desiccation and wilting occurred. Rehydration was subsequently applied, and post-rehydration recovery was evaluated by observing phenotypic restoration [14].

### 2.5. Transcriptome Sequencing Analysis

Transgenic line with optimal phenotypes and Col-0 seeds were sown in growth medium (vermiculite:nutrient soil = 1:1, *v*/*v*). After four weeks of growth, both Col-0 and the transgenic line were divided into two groups: one watered, as the control, and the other treated with 20% PEG-6000 solution to induce water deficit. Whole seedlings from both control and treated groups were collected 24 h post-treatment and immediately frozen in liquid nitrogen. Three biological replicates were prepared per treatment and sample, with each replicate comprising five uniformly grown individual plants. The samples were subjected to transcriptome sequencing at Personal Biotechnology Co., Ltd. (Shanghai, China), utilizing the NovaSeq 6000 platform (Illumina, San Diego, CA, USA). The difference expression of genes was analyzed by DESeq (v1.38.3) with screened conditions as follows: expression difference multiple |log_2_FoldChange| > 1, significant *p*-value < 0.05, using topGO (v2.50.0) to perform GO (Gene Ontology) enrichment analysis on the differential genes. ClusterProfiler (v4.6.0) software was used to carry out the enrichment analysis of the KEGG (Kyoto Encyclopedia of Genes and Genomes) pathway of differential genes.

### 2.6. Biochemical Indicators Determination

Following transcriptome sequencing sample collection, leaves from the remaining seedlings were harvested for biochemical indicators measurements. For each analyzed indicator, five seedlings per treatment and sample were processed, with collected leaf tissues immediately snap-frozen in liquid nitrogen for subsequent biochemical assays. The biochemical indicators detected include lignin content, methylglyoxal (MG) content, malondialdehyde (MDA) content, peroxidase (POD) activity, and glyoxalase I (Gly I) activity. All biochemical indicators were tested according to the instructions of the commercial reagent kit (Grace Biotechnology, Suzhou, China).

### 2.7. qRT-PCR Validation of Transcriptome Data

Seventeen up-regulated DEGs were selected to design primers for qRT-PCR validation. Reverse transcription was performed using the remaining RNA from transcriptome sequencing. First-strand cDNA synthesis and qRT-PCR were performed using the HiScript II Q RT SuperMix for qPCR (+gDNA wiper) kit (Vazyme, Nanjing, China) and SuperReal PreMix Plus (SYBR-Green) (Vazyme, Nanjing, China). The qRT-PCR reactions were performed with a Bio-Rad CFX96 Real-Time PCR Detection System using 1 μL cDNA as the template and *AtUBQ5* as the internal reference gene. The relative expression level was calculated using the 2^−ΔΔCt^ method with three technical replicates and three biological replicates. Data analysis was performed using GraphPad Prism 8.0.1.244. Primer sequences are detailed in Table S1.

### 2.8. Screening of Protein Interaction with GhA01EP1 by Glutathione-S-Transferase (GST) Pull-Down Assay and Liquid Chromatography Tandem Mass Spectrometry (LC-MS/MS) Analysis

The cDNA sequence of *GhA01EP1* was cloned into the GST-tagged fusion vector pGS21T. The constructed GST-*GhA01EP1*-pGS21T expression vector was transformed into the host bacterium *Escherichia coli* Rosetta (DE3). Positive cells were selected and inoculated into LB culture medium, cultured at 37 °C for approximately 6 h (OD600 = 0.6), and then isopropylthio-β-D-galactoside (IPTG) was added at a final concentration of 0.5 mmol·L^−1^ to induce the expression of the fusion protein. After induction at 37 °C for 2 h, the bacterial cells were collected and lysed, and the processed bacterial protein samples were subjected to 12% sodium dodecylsulfate-polyacrylamide gel electrophoresis (SDS-PAGE) to detect protein expression. If obvious expression bands were detected, the cells were further cultured.

Bacterial cells containing the GST-*GhA01EP1*-pGS21T plasmid (3000 mL) and GST-pGS21T plasmid (400 mL) were cultured to OD600 = 0.6, and expression was induced with 0.5 mmol·L^−1^ IPTG at 15 °C for 16 h. The cells were collected by centrifugation (6000 r·min^−1^, 5 min), and the supernatant was discarded. The precipitated cells were resuspended in 100 mL buffer D (20 mmol·L^−1^ Tris-HCl [pH 8.0], 50 mmol·L^−1^ NaCl, and 0.1% Triton-100), sonicated (600–800 W, 30 min) in an ice bath, then centrifuged (10,000 r·min^−1^, 15 min), and the supernatant was collected and loaded into the GST column (40–60 min). The GST medium was washed with 200 mL buffer E (20 mmol·L^−1^ Tris-HCl [pH 8.0], 500 mmol·L^−1^ NaCl, and 0.1% TritonX-100) and with 20 mL buffer D1 (20 mmol·L^−1^ Tris-HCl [pH 8.0], 50 mmol·L^−1^ NaCl, and 0.1% Triton-100).

Crude protein was extracted from *GhA01EP1*-transgenic *Arabidopsis* whole seedlings. Two 4-mL samples of the protein extract were incubated with GST-beads in combination with either the GST or GhA01EP1 proteins. After incubation, the solution was centrifuged and washed twice with 2.5 mL TNT Buffer (20 mmol·L^−1^ Tris [pH 8.0], 125 mmol·L^−1^ NaCl, and 0.5% TritonX-100), and then 100 µL of 2×loading buffer was added after the supernatant was discarded. The solution was mixed, boiled for 10 min, centrifuged (10,000 r·min^−1^, 2 min), and the supernatant was aspirated for SDS-PAGE. After staining, the protein band was cut from the gel for liquid chromatography/mass spectrometry analysis, and the proteins were detected with an AB Sciex TripleTOF 5600+ mass spectrometer. The data were processed with Mascot 2.3 software using the Uniprot protein database (https://www.uniprot.org).

### 2.9. Validation of Protein Interactions Using GST Pull-Down Assay

The prokaryotic expression vectors GST-*GhA01EP1*-pGS21T, His-TRX-*ALEU*-pET32T, and His-TRX-*SCPL35*-pET32T were constructed and transformed separately into *E. coli* BL21 (DE3) cells. The expression of the GST-fusion and His-fusion proteins were induced with IPTG, then the bacterial cells were lysed, and the fusion protein was extracted and purified. Purified ALEU (SCPL35) protein was incubated with GST-beads in combination with GST or GhA01EP1 for 4 h. After centrifugation (1000× *g*, 5 min), the supernatant was discarded, the pellet was washed three times with 2.5 mL TNT Buffer, then 100 μL of 2× loading buffer was added, and the solution was mixed, boiled for 10 min, and then centrifuged (10,000 r·min^−1^, 2 min). The supernatant was collected for Western blot detection. The Input group was not processed by incubation with GST-beads.

### 2.10. Validation of Protein Interactions Using Co-Immunoprecipitation (Co-IP) Assay

The constructed overexpression *GhA01EP1*-GST-p1301S, *ALEU*-GFP-p1301S, and *SCPL35*-GFP-p1301S vectors were separately transformed into *Agrobacterium* EHA105. Equal amounts of *Agrobacterium* cells harboring the *GhA01EP1*-GST-p1301S plasmid and those harboring the *ALEU*-GFP-p1301S (or *SCPL35*-GFP-p1301S) plasmid were injected separately into tobacco leaves as experimental controls. After incubation for 48 h in the dark and then in the light for 48–72 h, the protein was extracted from the tobacco leaf for Co-IP validation. Western blotting was performed using GST and GFP antibodies as the primary antibodies, and horseradish peroxidase-labeled sheep anti-rabbit immunoglobulin G was used as the secondary antibody.

## 3. Results

### 3.1. GhA01EP1 Promotes the Development of GhA01EP1-Transgenic Arabidopsis and Results in Earlier Flowering Time

**Figure 1:**
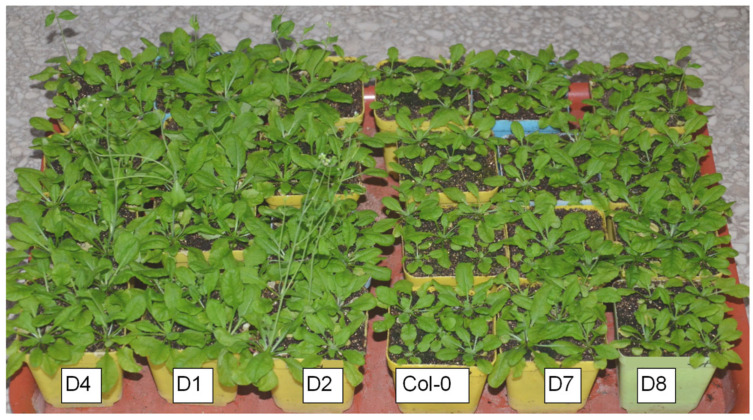
The Comparison of flowering time between transgenic lines and Col-0. D1, D2, D4, D7, and D8 represent the five transgenic T_3_-generation lines.

Under the water-sufficient treatment, five T_3_ transgenic lines grew faster than Col-0 and exhibited earlier flowering (Figure 1). The *GhA01EP1*-transgenic *Arabidopsis* demonstrates a mean reduction of 3.8 ± 0.5 days in days to flowering compared to Col-0. Among the five transgenic lines, the D2 line demonstrated optimal performance with the fastest developmental progression and earliest flowering time.

### 3.2. GhA01EP1 Affects GhA01EP1-Transgenic Arabidopsis Morphology and Seed Yield

**Figure 2:**
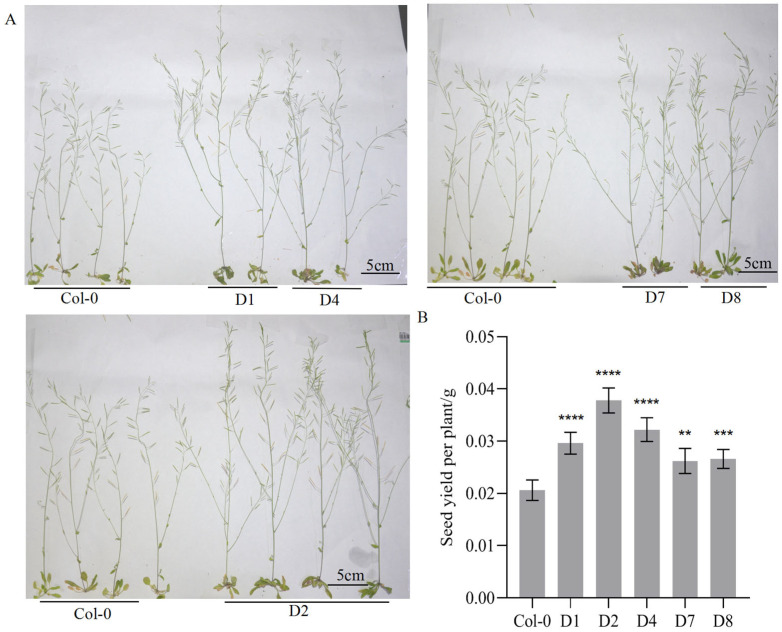
Effects of *GhA01EP1* on morphological traits and seed yield in transgenic *Arabidopsis*. (**A**) Comparison of primary stem height and branch number on the main stem between transgenic lines and Col-0 during later developmental stages. (**B**) Comparison of seed yields between transgenic lines and Col-0. Bars and error bars indicate the mean and standard deviation (n = 5). **, ***, and **** respectively represent a significant difference with *p* < 0.01, 0.001, and 0.0001.

During later developmental stages, transgenic lines exhibited greater main stem height and more branches compared to Col-0 (Figure 2A). Transgenic lines exhibited a significant increase of 1.13 ± 0.51 in branch number compared to Col-0 (*p* < 0.01). To assess the impact on seed yield, mature dried seeds from Col-0 and transgenic lines were collected and quantified. The seed yields of all transgenic lines were higher than those of the Col-0, showing an extremely significant difference (Figure 2B).

### 3.3. The GhA01EP1 Enhances Water Deficit Tolerance in GhA01EP1-Transgenic Arabidopsis

**Figure 3:**
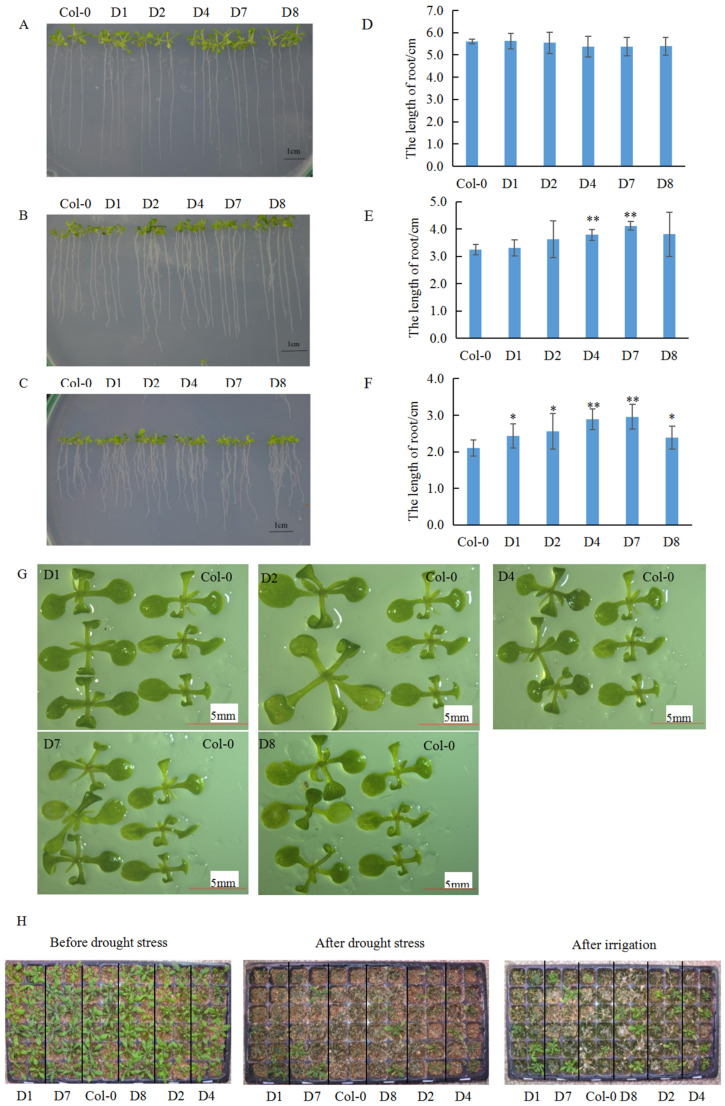
Comparative analysis of growth performance between transgenic *Arabidopsis* and Col-0 under water deficit stress. (**A**–**F**): The root length comparison of Col-0 and transgenic *Arabidopsis* under 0, 250, and 300 mmol·L^−1^ D-mannitol stress, respectively. (**G**): Comparison of rosette sizes between transgenic lines and Col-0 under 300 mmol·L^−1^ D-Mannitol stress. (**H**): Phenotypic alterations in transgenic *Arabidopsis* and Col-0 following water deficit and irrigation treatments. * and ** respectively indicate a significant difference with *p* < 0.05 and 0.01.

We conducted a comparative analysis of root length between Col-0 and transgenic lines under D-mannitol stress. Under 0 mmol·L^−1^ D-mannitol treatment, no significant differences in root length were observed between Col-0 and transgenic lines (Figure 3A,D). At 250 mmol·L^−1^ D-mannitol stress, D4 and D7 lines exhibited highly significant differences in root length compared to Col-0 (Figure 3B,E). Under 300 mmol·L^−1^ D-mannitol stress, D1, D2, and D8 lines showed significant differences, while D4 and D7 displayed extremely significant differences in root length relative to Col-0 (Figure 3C,F). Additionally, transgenic plants under 300 mmol·L^−1^ D-mannitol stress exhibited larger rosette sizes compared to Col-0 (Figure 3G).

In drought tolerance assays, ~3-week-old plants were subjected to 30 days of water deprivation, the result showed that transgenic lines experienced less severe stress symptoms than Col-0 (Figure 3H). After five days of rehydration, 33–50% of transgenic lines recovered normal growth, whereas Col-0 displayed only about 16.7% survival. These results confirm that *GhA01EP1* enhances water deficit tolerance in transgenic *Arabidopsis* [14].

To preserve the integrity of the article, the sections previously published in the *Journal of Cotton Science* are reproduced in this manuscript.

### 3.4. Transcriptome Analysis of Col-0 and GhA01EP1-Transgenic Arabidopsis

To further analyze the effects of *GhA01EP1* on the growth of transgenic *Arabidopsis*, the transcriptome of 4-week-old Col-0 (Col) and *GhA01EP1*-transgenic (EP1) *Arabidopsis* plants treated with 20% PEG-6000 (P) and water (CK) for 24 h. In the CK-EP1 vs. CK-Col comparison, 207 genes were up-regulated, and 21 genes were down-regulated. In the P-EP1 vs. P-Col comparison, 292 genes were up-regulated, and 34 genes were down-regulated. Thus, *GhA01EP1* affected the growth of *GhA01EP1*-transgenic *Arabidopsis* by predominantly up-regulating the expression of *Arabidopsis* genes. Furthermore, in the P-Col vs. CK-Col and P-EP1 vs. CK-EP1 comparisons, 549 and 412 genes were up-regulated, and 261 and 254 genes were down-regulated, respectively.

**Figure 4:**
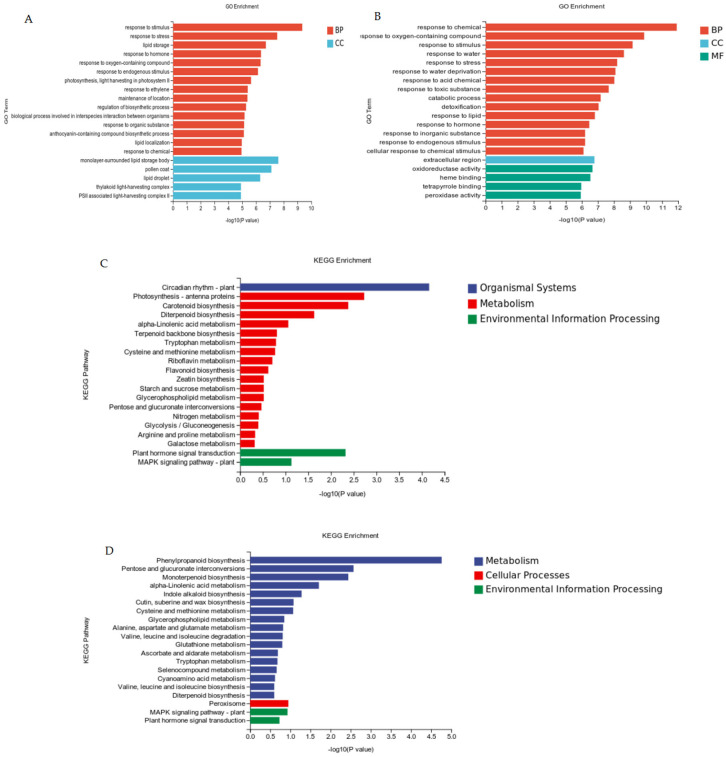
Enrichment of GO terms and KEGG pathways among DEGs in the CK-EP1 vs. CK-Col and P-EP1 vs. P-Col comparisons. (**A**) GO terms of the DEGs in the CK-EP1 vs. CK-Col comparison. (**B**) GO terms of the DEGs in the P-EP1 vs. P-Col comparison. (**C**) KEGG pathway enrichment analyses of the DEGs in the CK-EP1 vs. CK-Col comparison. (**D**) KEGG pathway enrichment analyses of the DEGs in the P-EP1 vs. P-Col comparison. The top 20 terms enriched in the biological process (BP), cellular component (CC), and molecular function (MF) categories, and the top 20 enriched KEGG pathways are shown.

Gene ontology (GO) term and Kyoto Encyclopedia of Genes and Genomes (KEGG) pathway enrichment analyses of the differentially expressed genes (DEG) were performed (Figure 4). In the CK-EP1 vs. CK-Col comparison, the DEGs were mainly involved in response to stimuli, stress, and hormones. Furthermore, the DEGs demonstrated significant involvement in photosynthesis and light harvesting in photosystem II. Cellular component analysis revealed that DEGs were predominantly enriched in monolayer-surrounded lipid storage bodies, pollen coats, and lipid droplets (Figure 4A). The DEG-associated metabolic pathways encompass circadian rhythm, photosynthesis, plant hormone signal transduction, the MAPK signaling pathway, terpenoid (carotenoid, diterpenoid, and terpenoid backbone) biosynthesis, and amino acid metabolism (tryptophan, cysteine, and methionine) (Figure 4C). In the P-EP1 vs. P-Col comparison, most DEGs are enriched in the pathways of biological processes. The main biological pathways include response to chemical, oxygen-containing compounds, stimuli, water, stress, water deprivation, acid chemicals, and so on; the molecular functions are mainly oxidoreductase activity, heme binding, tetrapyrrole, peroxidase activity; the cellular component is primarily localized to the extracellular region (Figure 4B). Among metabolic processes, the DEGs were mainly involved in phenylpropanoid biosynthesis, pentose and glucuronate interconversion, monoterpenoid biosynthesis, alpha-linolenic acid metabolism, peroxisome, plant hormone signal transduction, and the MAPK signaling pathway (Figure 4D).

Functional annotation of DEGs identified in the CK-EP1 vs. CK-Col comparison was performed using the UniProt database (Table S2). Multiple DEGs were functionally linked to reproductive development processes, including pollen tube development, pollen secondary cell wall development, tapetum development, pollen maturation and anther dehiscence, and maturation of male and female gametophytes. Notably, many genes were functionally annotated with circadian rhythm regulation and control of the photoperiodic flowering response. Others were linked to modulation of hormone metabolism pathways (auxin, cytokinin, abscisic acid [ABA], jasmonic acid [JA], salicylic acid [SA], and ethylene). Additional DEGs were associated with carbon-nitrogen allocation, lateral branch development, root development, disease resistance, and cold resistance. Up-regulation was observed for Gly I and oxidative stress-responsive genes, while specific DEGs were involved in broad-spectrum resistance.

### 3.5. Validation of RNA-Seq Results by qRT-PCR

**Figure 5:**
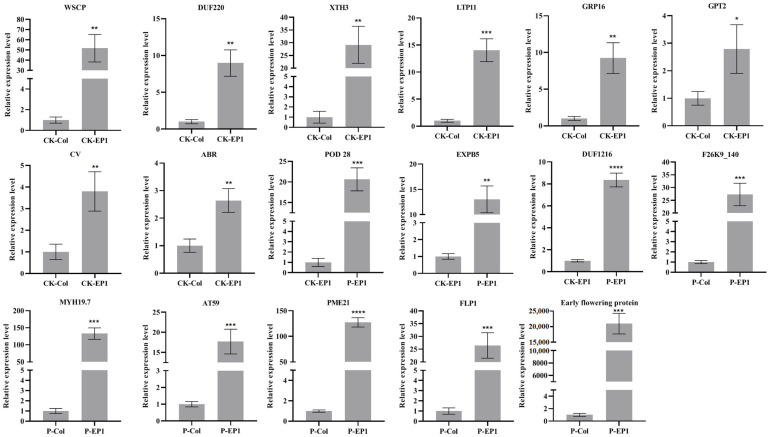
Validation of transcriptome data with qRT-PCR. CK: water treatment for 24 h, P: water deficit (20% PEG-6000 treatment for 24 h), EP1: transgenic line, Col: Col-0. AT1G72290: WSCP, AT3G25050: XTH3, AT5G07540: GRP16, AT4G31380: FLP1, AT3G21320: Early flowering protein. Bars and error bars indicate the mean and standard deviation. *, **, ***, and **** respectively represent a significant difference with *p* < 0.05, 0.01, 0.001, and 0.0001.

To validate RNA-seq results, seventeen up-regulated DEGs were analyzed by qRT-PCR (Figure 5). The results showed high consistency in these two methods, which indicated that the sequencing results were dependable. Among these seventeen genes, five floral development-associated genes (AT1G72290, AT3G25050, AT5G07540 from CK-EP1 vs. CK-Col comparison, AT4G31380, and AT3G21320 from P-EP1 vs. P-Col comparison) exhibited significant up-regulation, demonstrating *GhA01EP1*’s regulatory effects on floral development pathways in transgenic *Arabidopsis*.

### 3.6. Comparison of Biochemical Indicators Between Col-0 and GhA01EP1-Transgenic Arabidopsis

**Figure 6:**
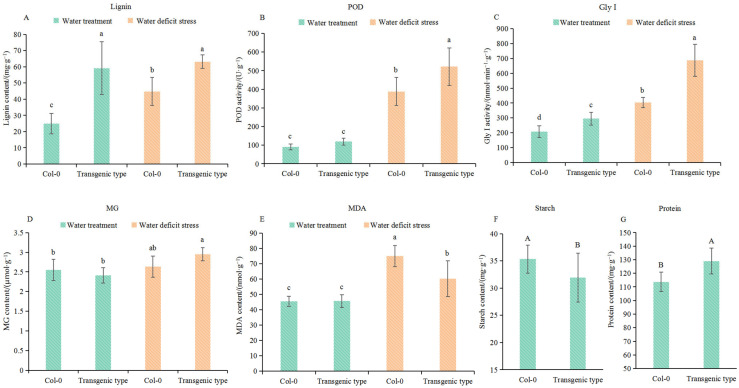
Comparison analysis of biochemical indicators of Col-0 and *GhA01EP1*-transgenic *Arabidopsis*. (**A**–**E**) Biochemical indicators in 4-week-old Col-0 and *GhA01EP1*-transgenic *Arabidopsis* plants grown under water treatment (CK) and water deficit stress (20% PEG-6000 treatment). Bars and error bars indicate the mean and standard deviation (n = 5). Different lowercase letters indicate significant difference (*p* < 0.05). (**F**,**G**) Seed protein and starch contents of Col-0 and *GhA01EP1*-transgenic *Arabidopsis*. Bars and error bars indicate the mean and standard deviation (n = 9). Different uppercase letters above bars indicate extremely significant difference (*p* < 0.01).

Based on the functions of the detected DEGs, an analysis of selected biochemical indicators of Col-0 and *GhA01EP1*-transgenic *Arabidopsis* grown under water and 20% PEG-6000 treatments was conducted (Figure 6). Under the water treatment (CK), the lignin content and Gly I activity in *GhA01EP1*-transgenic *Arabidopsis* were significantly higher than those in Col-0. POD activity, MG content, and MDA content were not significantly different between the transgenic line and Col-0. Under water deficit stress, the lignin content, POD, and Gly I activity in *GhA01EP1*-transgenic *Arabidopsis* were significantly higher than those in Col-0, whereas the MDA content was significantly lower. The transgenic line showed non-significantly higher MG content than Col-0.

Furthermore, the analysis of *Arabidopsis* seeds revealed significantly increased protein accumulation and reduced starch content in the *GhA01EP1*-transgenic line compared to Col-0 (Figure 6F,G).

### 3.7. Screening of Proteins Interacting with GhA01EP1 in GhA01EP1-Transgenic Arabidopsis

**Figure 7:**
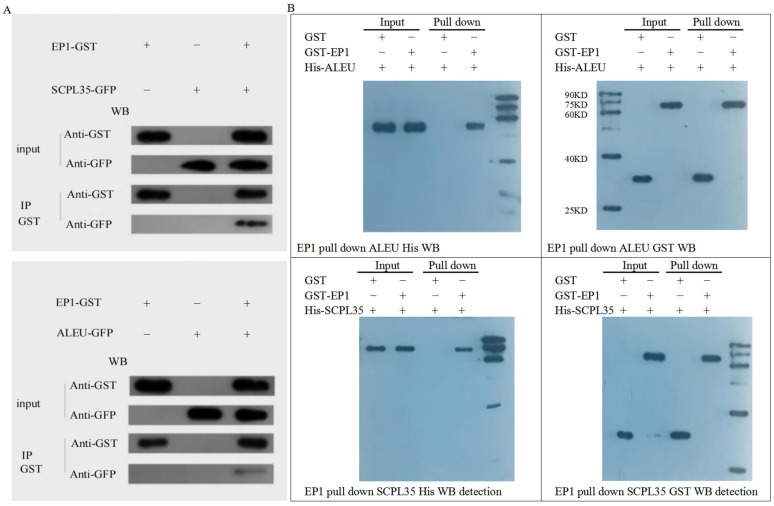
The validation of protein interactions through Co-IP and GST pull-down assays. (**A**) The interaction between the two candidate proteins and GhA01EP1 was validated by Co-IP assay. (**B**) The interaction between two candidate proteins and GhA01EP1 was validated by GST pull-down assay. EP1, GhA01EP1; GST, glutathione S-transferase; GFP, Green fluorescent protein; WB, Western blot; M: 90 KD, 75 KD, 60 KD, 40 KD, 25 KD bands.

To further investigate *GhA01EP1*’s role in *Arabidopsis* development, a GST pull-down assay identified 486 interacting proteins from *GhA01EP1*-transgenic *Arabidopsis*, among which 78 proteins with high reliability were selected for analysis (Table S3). SCPL35 (Q9LEY1) and ALEU (Q8H166) were selected for validation via Co-IP and GST pull-down based on their co-occurrence in both STRING v12.0 database predictions (https://cn.string-db.org/) and empirical interaction screens. The experimental results of the Co-IP assay (Figure 7A) and GST pull-down assay (Figure 7B) showed that SCPL35 and ALEU were both capable of interacting with GhA01EP1.

## 4. Discussion

In this study, compared with Col-0, *GhA01EP1*-transgenic *Arabidopsis* exhibited faster growth, earlier flowering, more branches, a higher seed yield, and stronger water deficit tolerance. To investigate the underlying mechanisms, we analyzed the transcriptomes of Col-0 and *GhA01EP1*-transgenic *Arabidopsis*, revealing that *GhA01EP1* regulates the expression of multiple genes. This section focuses on the function of DEGs in the CK-EP1 vs. CK-Col comparison.

### 4.1. Mechanism of GhA01EP1 in Enhancing Water Deficit Tolerance

Overexpression of *GhA01EP1* significantly up-regulated antioxidant stress-related genes, including AT5G58400, AT3G20340, AT1G80130, AT1G11210, AT4G25100, and AT2G43000. In addition to the above genes, genes participating in lignin biosynthesis (*TCF1*, AT3G55580) and cellular turgor pressure regulation (*JUB1*, AT2G43000) are also up-regulated. Ji et al. found that *TCF1* has been implicated in lignin synthesis [15]. Meanwhile, Wu et al. demonstrated that *JUB1* regulates hydrogen peroxide homeostasis, suppresses senescence, and coordinates proline and trehalose biosynthesis [16]. Both metabolites enhance water deficit tolerance by maintaining cellular osmotic balance.

*GhA01EP1* induces the up-regulation of *Gly I*, leading to elevated Gly I protein levels that modulate MG accumulation. MG functions as a signaling molecule under non-stress conditions [17]. Under abiotic stress, the MG levels surge rapidly, and excessive MG inhibits seed germination, photosynthesis, and root growth [18]. However, the plant glyoxalase system, comprising the Gly I, Gly II, and Gly III enzymes [19], can detoxify MG and confer stress tolerance [20]. Notably, heterologous overexpression of rice *Gly I* in *E. coli* and tobacco improved their adaptation to various abiotic stresses [21]. Wu et al. overexpressed *Gly I* in mustard, which improved the tolerance of mustard to salt, drought, and heavy metal stress [22]. In cotton, Xu et al. found that some members of the glyoxalase family would respond to high-temperature treatment. The protein interaction network of glyoxalases in *Gossypium hirsutum* implied that most members can participate in various life processes [23].

Theoretically, elevated Gly I activity in plants should correlate with reduced MG content. However, under water deficit stress in this study, *GhA01EP1*-transgenic *Arabidopsis* exhibited marginally higher (non-significant) MG levels compared to Col-0, aligning with observations by Hossain et al. [24]. Intriguingly, MG accumulation in pumpkin positively correlates with Gly I activity under various abiotic stresses. In several plant species—including mung bean, mustard, tomato, and rice—the increase in MG content is accompanied by a significant increase in the activities of Gly I and Gly II, indicating that stress-induced MG may be a signal to enhance the plant defense mechanism by increasing the ability to detoxify MG [24].

Beyond its detoxification role, *Gly I* has been shown to have multifaceted biological functions: (1) *Gly I* overexpression can increase plant yield [25,26]. (2) *Gly I* can mediate cell division and proliferation [27]. (3) *Gly I* can regulate pollen–pistil interactions to improve pollination efficiency [28].

To sum up, *GhA01EP1* can induce *Gly I* gene expression in *GhA01EP1*-transgenic *Arabidopsis* to enhance MG detoxification capacity, improve pollination efficiency, and promote cell division.

### 4.2. Mechanism of GhA01EP1 Regulation of Precocity and Rapid Growth of Plants

*GhA01EP1* up-regulates these genes associated with circadian rhythm regulation, photoperiodic flowering response, and flower development, including *LNK1* (AT5G64170), *GIGANTEA* (AT1G22770), *APRR5* (AT5G24470), *APRR7* (AT5G02810), and *TEM2* (AT1G68840). The *LNK1* has been proven to activate clock-controlled genes [29] and serves as a transcriptional coactivator for expression of the clock genes *PRR5* and *TOC1* [30]. Research has found that the GIGANTEA protein is involved in the regulation of circadian rhythm and photoperiodic flowering [31] and phytochrome B signaling [32]. Nakamichi et al. demonstrated that *APRR5* and *APRR7* can participate in the positive and negative feedback loops of the circadian clock [33]. In addition, *APRR7* is also involved in oxidative stress response and regulation of stomatal conductance [34]. *TEM2*, as a direct inhibitor of the flowering gene *FT*, is used to balance the expression of other flowering-related genes and ensure strict regulation of flowering time [35]. Moreover, numerous other DEGs (AT5G50800, AT5G46795, AT2G21650, AT1G72290, AT1G54560, AT3G61910, AT5G07530, AT1G18280, AT3G13890, and AT2G44810) are also involved in flower development, including gametogenesis, pollen tube and anther growth, pollen-stigma recognition, and ovule maturation. As a result, up-regulation of the above DEGs likely drives the accelerated flowering in *GhA01EP1*-transgenic *Arabidopsis*.

*GhA01EP1* modulates hormone metabolism-associated genes to accelerate plant maturation and growth. Jasmonates—including jasmonic acid (JA), methyl jasmonate, and jasmonoyl-isoleucine (JA-Ile)—enhance crop productivity, abiotic stress tolerance, and pest resistance [36]. In *GhA01EP1*-transgenic *Arabidopsis*, we observed up-regulated *AOC1* (AT3G25760) and *DAD1* (AT2G44810), both of which have been demonstrated to be involved in the synthesis of JA. Research shows that *AOC1* generates 12-oxo-phytodienoic acid (JA precursor), while *DAD1* encodes a phospholipase A1 catalyzing JA biosynthesis initiation, synchronizing pollen maturation, anther dehiscence, and floral opening [37]. Despite their benefits, excessive jasmonates induce leaf malformation, senescence, and lethality. Many genes in the CYP94 subfamily (cytochrome P450 family) oxidize JA-Ile to 12OH JA-Ile, maintaining optimal JA-Ile levels [38]. *GhA01EP1*-transgenic *Arabidopsis* exhibited elevated *CYP94B1* (AT5G63450) and *CYP94B3* (AT3G48520) expression, which mediate JA-Ile catabolism.

In *GhA01EP1*-transgenic *Arabidopsis*, *GhA01EP1* also up-regulates auxin metabolism-associated genes: *YUC5* (AT5G43890), *MYB73* (AT4G37260). Research indicates that *YUC5* encodes a monooxygenase critical for auxin biosynthesis [39], and *MYB73* is an auxin-responsive transcription factor activating *IAA19* expression [40]. In addition, two additional auxin-responsive protein genes (AT3G53250, AT4G22620) showed elevated expression. Collectively, these auxin metabolism-related DEGs likely accelerate *GhA01EP1*-transgenic *Arabidopsis* growth.

Additionally, the expression of DEGs responsive to cytokinin (AT3G16360) and salicylic acid signal transduction (AT5G47240) was up-regulated, while ethylene biosynthesis genes (AT4G37770, AT2G22810) were suppressed. In summary, *GhA01EP1* enhances plant growth through coordinated regulation of phytohormone metabolism pathways.

### 4.3. Mechanism of GhA01EP1 Influence on Yield Traits

*GhA01EP1* alters lateral branch development in *GhA01EP1*-transgenic *Arabidopsis* by modulating key regulatory genes, including *COL12* (AT3G21880), *CCD8* (AT4G32810), and *CCD7* (AT2G44990). Evidence reveals that *COL12* enhances rosette branching and reduces inflorescence height while regulating flowering [41]. Auldridge et al. and Brewer et al. found that the mutants max3 and max4 of *Arabidopsis* harbor mutations to *AtCCD7* and *AtCCD8*, respectively, and both are characterized by having highly branched phenotypes [42,43]. In the study, the combined suppression of *CCD7/8* and induction of *COL12* likely underlie the increased branching and elevated seed yield observed compared to Col-0.

*GhA01EP1* modulates carbon-nitrogen allocation in the *GhA01EP1*-transgenic line, a process governed by intricate genetic networks. The *QUA-QUINE STARCH* (*QQS*) is species-specific, and altered *QQS* expression in *Arabidopsis* affects carbon partitioning to starch and protein. Li et al. reported that *QQS* can increase the protein content and reduce the starch content in transgenic soybean, corn, and rice seeds [44]. In the present study, although *QQS* (AT3G30720) was down-regulated, the protein content was increased and the starch content was decreased in seeds of *GhA01EP1*-transgenic *Arabidopsis* compared with those of Col-0, indicating that *GhA01EP1* has a regulatory effect on carbon-nitrogen allocation in *GhA01EP1*-transgenic *Arabidopsis*. However, given the complexity of carbon-nitrogen allocation, the down-regulated expression of *QQS* may only balance the allocation process and avoid excessive protein accumulation.

### 4.4. Analysis of Interacting Proteins of GhA01EP1

SCPL35, a serine carboxypeptidase-like protein annotated as a secreted proteolytic enzyme in UniProt, shows limited characterization of its specific metabolic functions. With STRING v12.0 interaction network analysis (https://cn.string-db.org) identified CEP1 (Q9FGR9)—a programmed cell death regulator essential for tapetal degradation and pollen maturation [45]—among its 10 predicted interactors. This CEP1 interaction suggests SCPL35 and *GhA01EP1*’s potential involvement in the floral development of *GhA01EP1*-transgenic *Arabidopsis*. ALEU is a thiol protease, which may play a role in proteolysis, leading to mobilization of nitrogen during senescence and nitrogen starvation (as annotated in the UniProt database). NCBI BlastP analysis revealed 66% and 75.98% amino acid sequence identity between AtSCPL35 and AtALEU, respectively, and their putative ortholog in *G. hirsutum*.

To further validate the function of *GhA01EP1*, we have conducted CRISPR/Cas9-mediated gene editing and overexpression analyses in cotton. Currently, T_0_ generation plants carrying gene-edited constructs and overexpression cassettes have been successfully obtained. Subsequent functional validation will be performed using these transgenic cotton lines.

## 5. Conclusions

The present results indicate that the effects of *GhA01EP1* on the growth and development of *Arabidopsis* involve several metabolic pathways: (1) *GhA01EP1* promotes plant growth and early flowering by affecting the expression of genes associated with circadian rhythm regulation, photoperiodic flowering, and hormone metabolism; (2) *GhA01EP1* affects the regulation of the antioxidant system, glyoxalase system, and the synthesis of osmoregulatory substances and lignin, thereby improving plant water deficit tolerance; (3) *GhA01EP1* may increase the number of branches by regulating genes associated with branch development, thereby increasing the yield.

At present, cotton breeding strategies predominantly target individual trait enhancement, facing challenges in achieving multi-trait synergy due to inherent negative genetic correlations between stress resistance and yield traits. However, the exploitation of pleiotropic regulatory genes provides a viable approach to circumvent this limitation. In the study, *GhA01EP1* can simultaneously improve the precocity, water deficit resistance, and seed yield in transgenic *Arabidopsis*, which has an important theoretical basis for the synergistic improvement of excellent cotton traits.

## Data Availability

The data presented in this study are available in the article and Supplementary Materials.

## Supplementary Materials

The following supporting information can be downloaded at: https://www.mdpi.com/article/10.3390/genes16060669/s1, Table S1: Primer sequences for qRT-PCR; Table S2: The functional annotation of DEGs in the CK-EP1 vs. CK-Col comparison; Table S3: The 78 proteins that interact with GhA01EP1 were screened using a GST pull-down assay in *GhA01EP1*-transgenic *Arabidopsis*.

## Abbreviations

The following abbreviations are used in this manuscript:

Co-IP	Co-immunoprecipitation
Col	Columbia
DEGs	Differentially expressed genes
Gly	Glyoxalase
GO	Gene ontology
GST	Glutathione S-transferase
JA-Ile	Jasmonoyl isoleucine
KEGG	Kyoto Encyclopedia of Genes and Genomes
LC-MS/MS	Liquid chromatography tandem mass spectrometry
MDA	Malondialdehyde
MG	Methylglyoxal
POD	Peroxidase
QQS	Qua-quine starch
qRT-PCR	Quantitative real-time polymerase chain reaction
ROS	Reactive oxygen species
