# Supplementary material for: GhA01EP1 of Upland Cotton Stimulates Precocity, Improved Water Deficit Tolerance, and High Seed Yield in Transgenic Arabidopsis
Source: Genes (Basel). 2025 May 30;16(6):669. doi: 10.3390/genes16060669 (PMC12192239; doi:10.3390/genes16060669)
Supplement: Supplementary file 1 [file genes-16-00669-s001.zip › Supplementary files 1-Table S1.pdf]

Table S1 Primer sequences for qRT-PCR

Gene ID	Description	Forward primer	Reverse primer	Comparison
AT1G72290	WSCP	CTCATCATTCTCCTGTTTGCTGC	ACCACCTCCGTTTTTACTCTCG	CK-EP1 vs CK-Col comparison
AT1G23660	DUF220	GAGTCCAAAAGCCCTAAATCTGAA	CACCTTAGCAGGAGCATCATACC	
AT3G25050	XTH3	TTGATGCTAGGGTTTTCGGAGG	AACCCACCACCTGAAGATTGAT	
AT4G33355	LTP11	TACGATTTTATTGGGGATTGCG	CAGCACATTGGAGACGGATTTC	
AT5G07540	GRP16	TGCCGTAGCATCTGTAGTCTTCTT	CGGACTGAAGATGACAAAAAGCG	
AT1G61800	GPT2	CCATCTTCGTCTTCCTTCTCCA	GAAGCAGATGAAATGTGTAGAGGC	
AT2G25625	CV	TAACAATGGCAGGGAGAATAAGC	ACACTTGTTTTTCCGTGGCTCT	
AT3G02480	ABR	CCAAGGATGCTGCTGCTTCA	GGAAACAACATCAGAAAGTAGCAGG	
AT3G03670	Peroxidase 28	ACGACTGTTTTGTCCAGGGCT	CCTCTCACGCTAAAGTTTGGGC	P-EP1 vs CK-EP1 comparison
AT3G60570	EXPB5	AAGGGTTGTGGGGCTTGTTAC	TTTTAGTTCTCCAAGGTTGCGTAGT	
AT3G28810	DUF1216	ATTCGCCAAGTATGTCTGCCC	TCCAGCCTTGGCCTTCATGT	
AT3G62710	F26K9_140	TAATGGCGGCGGCAATGTTC	TGGAATATCCGCTTTGCTATGTC	
AT5G39880	MYH19.7	CGGCTCACGAAGCGAAAAAG	CACGGTTTTCTCCAAGTGCTCT	P-EP1 vs P-Col comparison
AT1G14420	AT59	GACAACTGCTGGCGATGCGA	TGTCCTAAGCCCTATGTGAGCCT	
AT3G05610	PME21	CTTCGTGGGTAGACCAGCGT	CGTCTTGTATTGACCGCTCCC	
AT4G31380	FLP1	TGTGGGTATTCAACAAAAACGG	CCTCATCCTCTGCTGCTGGTTA	
AT3G21320	EARLY FLOWERING protein	TGTATGTAAAAGACCGAGTCACCC	GGTATTGGTTGGTTAGGTCCGA	
Internal reference gene	AtUBQ5	CGTTGCCTCAAAAGATGCAGATC	ACATTGTCGATGGTGTCGGATG	
